# Nondestructive detection of lead chrome green in tea by Raman spectroscopy

**DOI:** 10.1038/srep15729

**Published:** 2015-10-28

**Authors:** Xiao-Li Li, Chan-Jun Sun, Liu-Bin Luo, Yong He

**Affiliations:** 1 College of Biosystems Engineering and Food Science, Zhejiang University, 866 Yuhangtang Road, Hangzhou 310058, China

## Abstract

Raman spectroscopy was first adopted for rapid detecting a hazardous substance of lead chrome green in tea, which was illegally added to tea to disguise as high-quality. 160 samples of tea infusion with different concentrations of lead chrome green were prepared for Raman spectra acquirement in the range of 2804 cm^−1^–230 cm^−1^ and the spectral intensities were calibrated with relative intensity standards. Then wavelet transformation (WT) was adopted to extract information in different time and frequency domains from Raman spectra, and the low-frequency approximation signal (ca4) was proved as the most important information for establishment of lead chrome green measurement model, and the corresponding partial least squares (PLS) regression model obtained good performance in prediction with R_p_ and RMSEP of 0.936 and 0.803, respectively. To further explore the important wavenumbers closely related to lead chrome green, successive projections algorithm (SPA) was proposed. Finally, 8 characteristic wavenumbers closely related to lead chrome green were obtained and a more convenient and fast model was also developed. These results proved the feasibility of Raman spectroscopy for nondestructive detection of lead chrome green in tea quality control.

Green tea is one of the six major teas in China with the longest history, the highest output and the widest sphere of consumption. Among all the sensory evaluation indexes of green tea, color plays a particularly important role. Color is not only the most intuitive impression but also closely related to liquor color, aroma, taste^1^ and even antioxidant activity of green tea^2^. In recent years, the media frequently exposed that some peddlers illegally added lead chrome green into tea to fake a wonderful color for economic exploitation^3^. The lead chrome green is a type of powder dye with a light green color, consisting of lead chrome yellow and phthalocyanine blue or prussian blue pigments^4^,^5^, which are harmful to human health. It has been banned to add any colorant in tea production in China. However, there is still no standard method for detection of lead chrome green in tea.

At present, the existence of lead chrome green in tea is arbitrarily inferred based on the existence of lead and chromium^3^,^6^,^7^. However, soil heavy metal pollution and vehicle exhaust emissions may also lead to the accumulations of lead and chromium in tea in tea production process. Therefore, the existence of lead or chromium cannot prove the existence of lead chrome green. In addition, the traditional method for detection of lead and chromium, depending on chemical process, is very labor-intensive and time-consuming including a series of complicated procedures such as extraction, digestion, heating, cooling and so on. Raman spectroscopy works on a molecular level, reflecting the information of molecular vibration and rotation^8^. Recently, Raman spectroscopy as a nondestructive and time-efficient method has been used for qualitative detection of mineral dyestuffs^9^, widely employed in mineralogy and archaeology^10^,^11^,^12^. Wang *et al.*^13^ characterized the chemical structures of late Permian coals with four types from Southern China by Raman spectroscopy. Holakooei *et al.*^14^ investigated the components of different colors in a pre-seventeenth century wall painting using micro-Raman spectroscopy. Besides, researches on quantitative detection of heavy metal ions, such as lead and chromium ions, by Raman spectroscopy have also been carried out^15^,^16^. Wang *et al.*^17^ developed a surface enhanced Raman scattering (SERS) DNAzyme biosensor for the detection of Pb ion. Ji *et al.*^18^ provided a facile method for the detection of Cr (VI) in aqueous solutions based on semiconductor-enhanced Raman spectroscopy. These researches successfully proved the potential of Raman spectroscopy for detection of lead and chromium ions. However, these methods can only detect a particular ion (lead or chromium) and they cannot simultaneously detect all the components of lead chrome green, which consists of lead chrome yellow and phthalocyanine blue or prussian blue pigments, as well as other additives. Lead chrome green is a mixture and the detection of a particular chemical substance cannot be used as the detection criterion of lead chrome green, and there is still no national standard method for detection of lead chrome green. Furthermore, there is no report to rapid and nondestructive quantitative detection of lead chrome green in tea based on Raman spectroscopy. In this study, Raman spectroscopy was first applied to measure lead chrome green in tea quantitatively.

The main difficulties for Raman quantitative detection include the self-absorption of samples, the changes of refractive index caused by different concentrations of samples, the background noise from solvent and so on^19^. Therefore, it is difficult to determine the intrinsic Raman intensity which is proportional to the concentration of test object with so many influencing factors^20^. So, standards should be first measured to obtain the quantitative information. In this research, two different relative intensity standards were adopted and compared to correct the Raman spectral data.

Spectra obtained from Raman spectrometer often contain hundreds or thousands of spectral information, among which, parts of the information may correlate with the noise and background, and parts of the information may appear to be non-specific to the target component. These interfered information should be eliminated and the target information should be excavated to improve the predictive ability of the detection model. Therefore, chemometrics methods, which play a very important role in spectral data analysis, were applied for establishment of detection model and selection of characteristic wavenumbers.

The objectives of this study were: (1) to establish a reliable model for measurement of lead chrome green in tea based on Raman spectroscopy; (2) to select characteristic Raman wavenumbers for a convenient and fast measurement.

## Results and Discussion

### Detection of color

The main purpose of adding lead chrome green into tea was to fake high-grade tea with attractive color, however, the effect of adding amount for color change had no quantitative analysis. Therefore, the color of tea infusion with different concentrations of lead chrome green should be first investigated before the quantitative detection. Table 1 shows the color differences among different concentrations of lead chrome green detected by a spectrocolorimeter. The first row and column in Table 1 represent the concentrations of lead chrome green in tea, and the contents reflect the ΔE^*^ab values between two concentrations.

Generally speaking, color difference can be distinguished by naked eye when 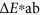 value is more than 1.5^21^. As seen in Table 1, the values in the second column are all beyond 1.5, which means that it will lead to an obvious color difference when the adding amount is greater than 2 mg/g. The obvious color difference caused by small amount of lead chrome green addition indicated a strong dyeing ability of lead chrome green and that was one of the main reasons why lead chrome green was chosen to fake tea color. Therefore, Raman spectroscopy was further used to detect the concentration of lead chrome green in tea.

### Qualitative identification of lead chrome green based on Raman spectra

**Figure 1:**
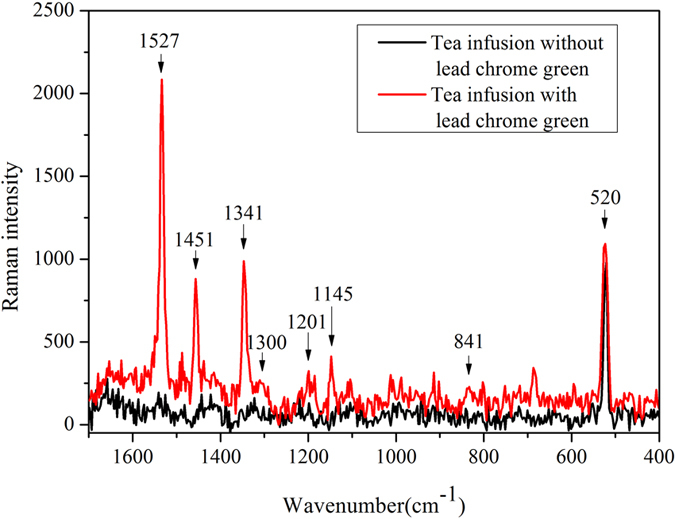
Raman spectra of samples.

Lead chrome green is a mixed colorant, mainly consist of lead chrome yellow and phthalocyanine blue or prussian blue. It is essential to first identify the components of lead chrome green. Figure 1 shows Raman spectra of tea infusion with or without lead chrome green from 1700 cm^−1^ to 400 cm^−1^.

As seen in Fig. 1, comparing with the spectrum of tea infusion without lead chrome green, there are many obvious peaks in the spectrum of tea infusion with lead chrome green, which can be inferred that these Raman peaks are caused by lead chrome green. Since these Raman peaks does not belong to the characteristic bands of prussian blue^22^, it can be concluded that the lead chrome green does not contain prussian blue. It is obvious to see that a peak at the wavenumber of 520 cm^−1^ is presented in both tea infusions with or without lead chrome green, this peak belongs to silicon, since the sample was placed on a silicon wafer. The peak at 841 cm^−1^ can be referred as the fingerprint of PbCrO_4_, as reported by Desnica^22^. The chemical structural formula of phthalocyanine blue contains plenty of chemical bonds of C-N, C-C, C-H and C = C. Both the characteristic peaks of C-N symmetric stretching and C-N symmetric bending (1200–1130 cm^−1^)^23^ are appeared in the spectrum in Fig. 1, which locate at the wavenumbers of 1145 cm^−1^ and 1201 cm^−1^. The peaks around 1300 cm^−1^ are associated with C-C stretching vibration^24^. Bands between the wavenumbers of 1290 cm^−1^ and 1370 cm^−1^ can be inferred as the vibrations of aromatic ring^24^, which is consistent with the structural formula of phthalocyanine blue. The peak at 1451 cm^−1^ can be attributed to C-H vibration^25^. The spectral feature at the wavenumber of 1527 cm^−1^ is considered to be the representation of C = C vibration in porphyrin ring^26^. The attribution analysis of these Raman peaks proved that the lead chrome green used in this research consisted of lead chrome yellow and phthalocyanine blue. Furthermore, it can be concluded that there is obvious difference of Raman spectral response between tea infusion with and without lead chrome green, and Raman spectra can probe the inherent vibrations of lead chrome green, these vibrations can be regarded as specific fingerprints for qualitative classification of tea infusion with or without lead chrome green.

### Quantitative detection of lead chrome green based on Raman spectra

#### Relative intensity correction

Since the difficulties of quantitative detection by Raman spectroscopy mentioned in the introduction, relative intensity standards were proposed to correct the data to obtain the quantitative information. The integrated intensity from 2804 cm^−1^ to 230 cm^−1^ and the intensity at the wavenumber of 520 cm^−1^ were respectively selected as the standards, and the spectral intensity ratios between the intensities of samples and that of the standards were used for quantitative analysis in this research. PLS was proposed to evaluate the results of corrections based on different relative intensity standards. The PLS model of the data without any correction was first built as a reference, successively, PLS models based on the data calibrated with the integrated intensity from 2804 cm^−1^ to 230 cm^−1^ and the intensity at the wavenumber of 520 cm^−1^ were respectively built. In general, an evaluation of a PLS model mainly depends on the values of R and RMSE. R represents the fitting degree of the model and RMSE reflects the deviation between the true values and the predicted values. The higher R (closer to 1) and lower RMSE the model obtains, the better results the model acquires. In addition, a small difference of R values in different sets (calibration, validation and prediction sets) means high stability of the model. The results of the models are represented in Table 2.

As shown in Table 2, R_p_ and RMSEP of model 1 were 0.932 and 0.817, respectively. In comparison with model 1, model 2 obtained a better result. On one hand, R_p_ rised from 0.932 to 0.950 and RMSEP reduced to 0.715 in the prediction set, on the other hand, the differences of performance among calibration, validation and prediction sets reduced. The outstanding performance of model 2 may attribute to the wonderful correction ability of full Raman spectral range of 2804 cm^−1^ to 230 cm^−1^. Model 3 obtained lower R and higher RMSEs comparing with model 2. The reason for the poor performance of model 3 may refer to the uncertain distance existed between the focal plane of the sample and the silicon wafer, leading a biased ratio of the spectral intensity between sample and silicon. Therefore, the data corrected by the integrated intensity was used in the following process.

#### Extraction of key information of Raman spectra based on wavelet transform (WT)

**Figure 2:**
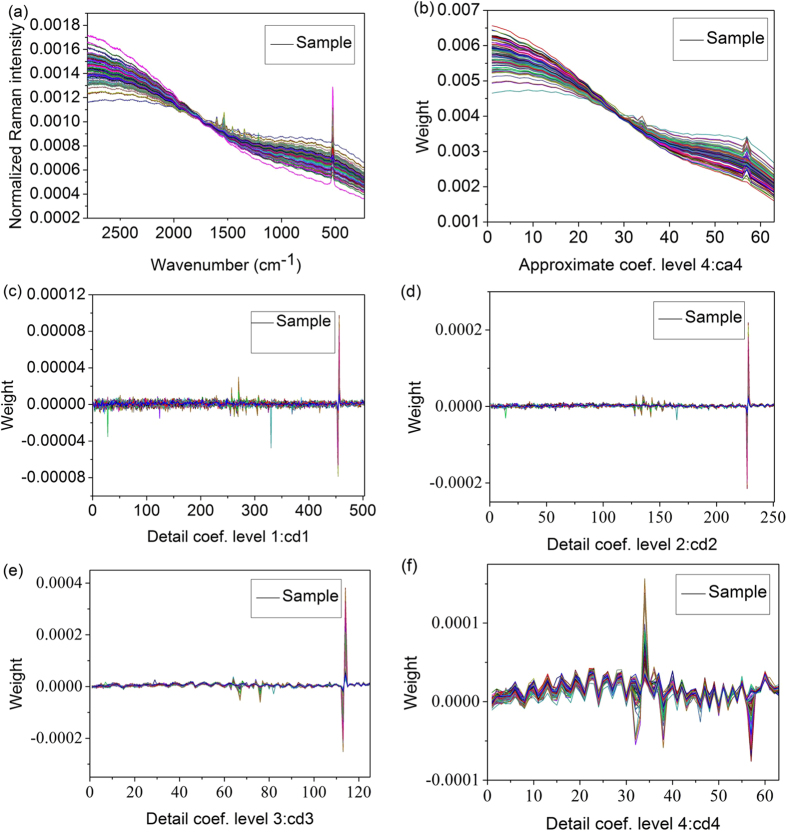
Wavelet decomposition coefficients. (**a**) normalized spectra, (**b**) approximate coefficient on level 4, detailed coefficient on (**c**) level 1, (**d**) level 2, (**e**) level 3, (**f**) level 4.

**Figure 3:**
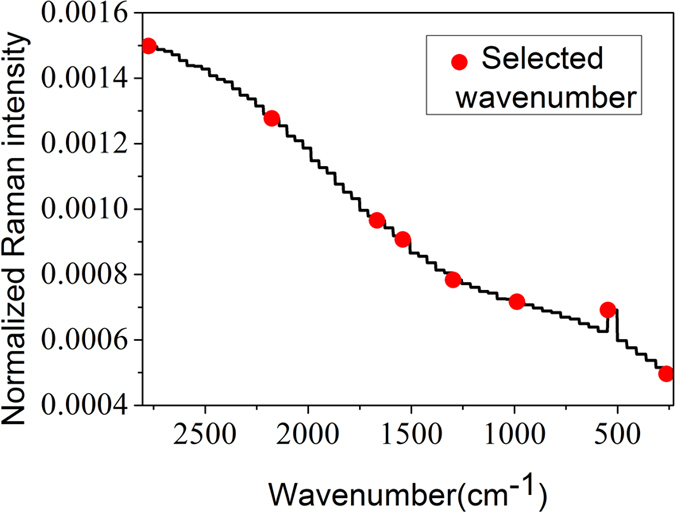
Distribution of the characteristic wavenumbers.

To further explore the detailed information of Raman spectra relating to lead chrome green in tea infusion, wavelet decomposition was used to build the detection model. In this study the spectra (S) were decomposed into five parts (cd1, cd2, cd3, cd4 and ca4) on four levels. On the first level, the spectra (S) were departed into two parts of wavelet coefficients through low-pass filter and high-pass filter, obtaining the approximate part (ca1) and the detailed part (cd1), respectively. In the next turn of decomposition, the approximate part was once again divided into two parts and that cycle repeated. After the decomposition, each sample was represented by five groups of wavelet coefficients as shown in Fig. 2(b–f). It can be found that the general trend of ca4 was similar to the original Raman spectra shown in Fig. 2(a). Figure 3(c–f) show the wavelet coefficients of the detailed parts on four levels and much high-frequency information could be found. After being processed by wavelet transform, the key information is mainly concentrated on approximate part^27^,^28^, so the wavelet approximate coefficients of ca4 were taken as the characteristic information of lead chrome green for further analysis.

To evaluate the impact of WT on the data in detail, the 63-dimensional wavelet coefficients of ca4 was set as independent variables to develop determination model based on PLS and the results were listed in Table 3. As shown in Table 3, model 4 obtained satisfactory performance with R_p_ and RMSEP values of 0.936 and 0.803, respectively. Furthermore, comparing with the results of model 2, the differences of R values among calibration, validation and prediction sets in model 4 decreased, which indicated that the stability of the model was improved. In general, comparing with model 2, model 4 obtained comparable accuracy and better stability, which demonstrated that WT was a useful tool in excavating the characteristic information and removing the irrelevant information of noise signal.

#### Selection of characteristic wavenumbers of Raman spectra

The key information of lead chrome green, represented by 63-dimensional wavelet low-frequency coefficients, had been extracted by WT. However, the wavelet coefficient of ca4 was dimensionless, since it was derived from the original spectral data by mathematic method. Although the linear relationship between wavelet coefficient and the concentration of lead chrome green in tea had been established by PLS model, the characteristic Raman peaks of chemical bonds in the samples were obscure. Therefore, the chosen wavelet coefficient of ca4 was used to reconstruction. By inserting the wavelet coefficient of ca4 into its initial position in the transformed vector and then setting the other coefficients to zero, following an inverse wavelet transformation, A4 was reconstructed based on ca4. To evaluate the performance of signal reconstruction, PLS model 5 was built based on the reconstructed spectra of A4 and the results were listed in Table 3. As seen in Table 3, model 5 obtained comparable results as model 4, besides, the dimension of the independent variables was resized to 1005. On the whole, it can be concluded that the signal reconstruction based on ca4 not only obtained the outstanding performance in PLS modeling, but also made a convenience for the following characteristic wavenumbers selection.

**Figure 4:**
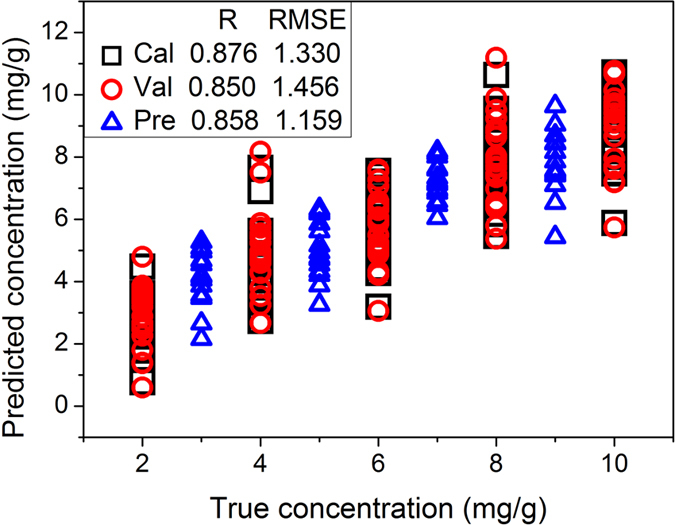
Scatter plot of true vs. predicted concentrations of lead chrome green by PLS model based on 8 characteristic wavenumbers.

For a rapid online detection system of lead chrome green in tea, the variables used in the detection model need to be simplified. Therefore, successive projections algorithm (SPA) was proposed to select the characteristic wavenumbers based on the low-frequency reconstructed spectra of A4 of the calibration set. Figure 3 shows the distribution of the selected 8 wavenumbers by SPA, and the corresponding characteristic wavenumbers were 2775, 2176, 1666, 1541, 1297, 988, 547 and 262 cm^−1^. On the basis of the 8 characteristic wavenumbers selected from the calibration set, detection model was built by PLS. Then the validation set was used to validate the model by full cross validation method and the prediction set was used to verify the prediction ability of the model. The results of PLS models based on the 8 characteristic wavenumbers are shown in Fig. 4. As seen in Fig. 4, the R values of validation and prediction sets were close and this phenomenon indicated that the performance of the model based on 8 characteristic wavenumbers was relatively stable. Furthermore, the number of variables reduced from 1005 to 8, which significantly improved the detection efficiency. The limit of detection (LOD) of lead chrome green was assessed by using the three times of standard deviation of the lowest lead chrome green concentration and the corresponding LOD of this method was 0.651mg/g.

### Analysis of the characteristic wavenumbers

Raman spectroscopy works on a molecular level, the spectral intensity at each wavenumber reflects the information of vibration and rotation of a certain molecular. The assignments of the 8 characteristic wavenumbers are listed in Table 4. As seen in Table 4, *λ*_2775_ and *λ*_2176_ are associated with H_2_PO_4_^−^, which exists in metal salt^29^. *λ*_1666_, *λ*_1541_ and *λ*_1297_ are assigned to phthalocyanine blue^24^,^26^,^30^, meanwhile, *λ*_988_ and *λ*_547_ are ascribed to lead chromate yellow^31^,^32^. *λ*_262_ is the characteristic Raman peak of calcite^33^. It is obvious to see that parts of the wavenumbers analyzed in section 2.2 were selected to be the characteristic wavenumbers by chemometrics methods, such as *λ*_1541_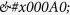 and *λ*_1297_. Meanwhile, several new appeared wavenumbers were selected in the process of characteristic wavenumbers selection, the assignments of these wavenumbers were some trace components in lead chrome green, as shown in Table 4. However, these newly selected wavenumbers were not significant in the spectral curve (Fig. 1) and this phenomenon may be due to the strong interference from fluorescence, which covered these closely related information. However, the combination of WT and SPA could solve this problem well and the availability of the corresponding detection model was also verified.

## Conclusions

This research proposed a novel method for determination of lead chrome green in tea based on Raman spectroscopy. First, the lead chrome green could be qualitatively identified based on the fingerprint Raman peaks of its compositions (lead chrome yellow and phthalocyanine blue). And the relative intensity standard method based on the integrated intensity of full range (2804 cm^−1^–230 cm^−1^) was proved as an effective way for quantitative detection of lead chrome green in tea. Additionally, the WT was proved to be a useful tool in extraction of key information of Raman spectra, and the model based on the wavelet approximate coefficients (ca4) achieved satisfactory prediction results with R and RMSE of 0.936 and 0.803, respectively. Finally, SPA was used to select the characteristic wavenumbers and 8 wavenumbers were obtained. In general, Raman spectroscopy was proved to be a useful technique for detection of lead chrome green and the 8 characteristic wavenumbers made a convenience and rapid detection of lead chrome green in tea quality monitoring.

## Materials and Methods

### Sample preparation

LongJing tea (purchased from Hang Zhou Yi Jiang Nan Tea co., LTD, Hangzhou, China) with 1 g was respectively mixed with 0, 2, 4, 6, 8 and 10 mg lead chrome green (purchased from Guang Zhou Hu An Pigment co., LTD, Guangzhou, China) in a beaker. Successively, 50 ml boiling water was poured into the beaker, soaking for 5 minutes. Then, the tea infusion was poured into a glass container for color measurement.

As for the acquisition of Raman spectra, tea with 9 dosages of lead chrome green (2, 3, 4, 5, 6, 7, 8, 9 and 10 mg/g) were prepared and soaked according to the above steps, 20 duplications were made for each dosage of 2, 4, 6, 8 and 10 mg/g, and 15 duplications were made for each dosage of 3, 5, 7 and 9 mg/g. Then, 45 ml tea infusion was taken into a centrifuge tube, centrifuging for 5 minutes at the rotational speed of 5000 rpm. Successively, 43.5 ml supernatant was discarded by a pipette and the remaining was oscillated for 20 s by an ultrasonic cleaner (KQ-500B, Kun Shan ultrasonic instrument co., LTD, Suzhou, China). Thus, sample was obtained for Raman spectroscopy scanning.

### Color measurement

A spectrocolorimeter (CM-600d, Konica Minolta, Japan) with detection mode of SCI (specular component include), was used to measure the color of sample. CIEL^*^a^*^b^*^ (CIELAB), which is considered as the most complete color model^7^, was used to describe the colors. In this study, 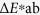 was used as the index to detect the relative perception difference between two colors. The computational formula of ΔE^*^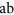 is shown as equation (1). There are three parameters in the model: 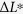 represents the brightness of color (negative value favors black, while positive value favors bright), 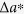 represents the color between red and green (negative value favors green, while positive value favors red), 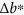 represents the color between yellow and bule (negative value favors blue, while positive value favors yellow)^34^.


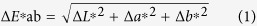


### Raman spectra acquisition

Sample with volume of 20 μl was placed to a silicon wafer by a pipette, following they were placed on a glass slide, and fixed under the 20x microscope objectives. Then, Raman spectra were collected with a Renishaw microscopic confocal Raman spectrometer (inVia-Reflex 532/XYZ, UK) equipped with a 532 nm laser source, 25 mv laser power. The exposure time and the number of accumulation were set as 1s and twice, respectively. The spectral range was from 2804 cm^−1^ to 230 cm^−1^ with a resolution of 2 cm^−1^. For each sample, the spectra of 15 uniformly distributed sampling points on the diagonal line in the field of vision were collected and averaged as a Raman spectrum of the sample.

### Sample division

Before establishing a detection model, all the samples were divided into three categories: calibration set, validation set and prediction set to further evaluate the model. The samples with concentrations of 2, 4, 6, 8 and 10 mg/g were chosen as the calibration samples and the left samples with concentrations of 3, 5, 7 and 9 mg/g were subsumed into the prediction set, meanwhile, the calibration set was validated by full cross validation method. Then calibration, validation and prediction sets obtained 100, 100 and 60 samples respectively in the end.

### Data analysis

Wavelet transform (WT) is the local analysis of time and space frequency, by the operations of stretch and translation, multiscale analysis of signals (functions) is achieved^35^,^36^. In the high frequency, time is subdivided, while in the low frequency, frequency is subdivided^37^. WT can automatically adapt to the requirements of time-frequency signal analysis, thus can focus on any detailed signal^38^,^39^. Due to the excellent function of local analysis, WT was applied to remove the background and noises for modeling. The computations were conducted in the Matlab 2010b.

Partial least squares (PLS) algorithm is a multivariate statistical analysis method, which can realize regression modeling, data structure simplification and correlation analysis simultaneously in an algorithm^40^,^41^. PLS not only maximizes the variance of the main components for more comprehensive information, but also makes the largest degree of correlation between independent and dependent variables for a sufficient use of the linear relation^42^. In this study, PLS algorithm was used to build the detection model of lead chrome green. The computations were operated with the “The Unscrambler V10.1” (CAMO PROCESS AS, Oslo, Norway).

Successive projections algorithm (SPA) is a selection method for sensitive wavenumbers. The variable set with the minimum redundancy could be selected from the spectral information, eliminating the collinearity between variables effectively with the least number of variables^43^. SPA was proposed here to reduce the complexity of model, making a convenience and rapid detection of lead chrome green. The detailed description of SPA can be found in the literature^44^,^45^. The computations of SPA were implemented in the Matlab 2010b.

## Additional Information

**How to cite this article**: Li, X.-L. *et al.* Nondestructive detection of lead chrome green in tea by Raman spectroscopy. *Sci. Rep.* **5**, 15729; doi: 10.1038/srep15729 (2015).

## Figures and Tables

**Table 1:** Color differences among different concentrations.

ΔE^*^ab	0	2 mg/g	4 mg/g	6 mg/g	8 mg/g
2 mg/g	1.681				
4 mg/g	2.431	0.854			
6 mg/g	2.495	1.236	0.632		
8 mg/g	3.112	1.782	0.998	0.618	
10 mg/g	3.283	2.180	1.499	0.951	0.603

**Table 2:** Results of PLS models based on the data calibrated with different relative intensity standards.

Model	Relative intensitystandard	Calibration set		Validation set		Prediction set	
		R_c_	RMSEC	R_v_	RMSECV	R_p_	RMSEP
Model 1	No	0.945	0.904	0.927	1.036	0.932	0.817
Model 2	Integrated intensity from 2804 cm^−1^ to 230 cm^−1^	0.950	0.865	0.937	0.966	0.950	0.715
Model 3	Intensity at the wavenumber of 520 cm^−1^	0.948	0.876	0.933	0.993	0.946	0.752

**Table 3:** Results of PLS models based on WT.

Model	Independentvariable	Dimension	Calibration set		Validation set		Prediction set	
			R_c_	RMSEC	R_v_	RMSECV	R_p_	RMSEP
Model 4	ca4	63	0.948	0.883	0.934	0.991	0.936	0.803
Model 5	A4	1005	0.947	0.886	0.933	0.994	0.935	0.809

**Table 4:** Assignment of the characteristic wavenumbers.

Wavenumber (cm^−1^)	Functional group/Chemical bond	Material
*λ*_2775,_ *λ*_2176_	H_2_PO_4_^−^	Metal salt^29^
*λ*_1666_	C=N	phthalocyanine blue^30^
*λ*_1541_	C=C	phthalocyanine blue^26^
*λ*_1297_	C-C	phthalocyanine blue^24^
*λ*_988_	SO_4_^2−^	lead chromate yellow^31^
*λ*_547_	Al-OH	lead chromate yellow^32^
*λ*_262_	CO_3_^2−^	Calcite^33^
